# PLCɛ maintains the functionality of AR signaling in prostate cancer via an autophagy-dependent mechanism

**DOI:** 10.1038/s41419-020-02917-9

**Published:** 2020-09-02

**Authors:** Zhen Quan, Ting Li, Yang Xia, Jiayu Liu, Zhongbo Du, Chunli Luo, Yunfeng He, Xiaohou Wu

**Affiliations:** 1 Department of Urology, The First Affiliated Hospital of Chongqing Medical University, 400016 Chongqing, China; 2 Key Laboratory of Laboratory Medical Diagnostics, Chongqing Medical University, 400016 Chongqing, China; 3 Department of Urology, The Second Affiliated Hospital of Chongqing Medical University, 400010 Chongqing, China

**Keywords:** Prostate cancer, Cell signalling

## Abstract

Androgen receptor (AR) signaling is a major driver of prostate cancer (CaP). Although most therapies targeting AR are initially effective in CaP patients, drug resistance is inevitable, mainly because of the inappropriate re-activation of AR pathway. However, the underlying mechanisms remain largely unknown. Here, we found that phospholipase C epsilon (PLCɛ) was highly expressed in CaP samples, and was closely associated with AR signaling activities. PLCɛ depletion triggered enhanced autophagic activities via AMPK/ULK1 pathway, causing autophagy-mediated AR degradation and inhibition of AR nuclear translocation. This subsequently reduced AR signals in CaP and inhibited AR-driven cell migration/invasion. Furthermore, a positive correlation between PLCɛ and AR signaling activity was also observed in bicalutamide-resistant CaP samples and in AR-antagonist-resistant CaP cell models. PLCɛ depletion resulted in the failure to establish AR-antagonist-resistant CaP cell lines, and hindered the metastatic prowess of already established ones. These findings suggest that PLCɛ-mediated autophagic activity alteration is indispensible for the functionality of AR signaling and for CaP development.

## Introduction

Prostate cancer (CaP) is one of the most common types of malignancies in males, ranking first in estimated new cancer cases and third in cancer-related deaths among males in the US^1^. Androgen receptor (AR) pathway is critically necessary in the development of CaP. Overactivation of AR signaling promotes cell proliferation, migration, and epithelial to mesenchymal transition (EMT), favoring tumorigenesis, and tumor progression^2^. Persistent activity of AR signaling has been observed in CaP as well as in castration-resistant prostate cancer (CRPC)^3,4^. Therefore, therapeutics targeting the AR signaling have been extensively explored and widely used in clinical practice, but drug resistance keeps emerging. It is urgent to understand how CaP cells “manage” to maintain a high level of AR signals.

Phospholipase C epsilon (PLCɛ) is a member of the PLC family of enzymes. All PLC enzymes possess the enzymatic activity of catalyzing hydrolysis of phosphatidylinositol 4,5-bisphosphate (PIP2) to generate two second messengers: inositol 1,4,5-trisphosphate (IP3), a regulator of intracellular Ca2+ level, and diacylglycerol (DAG), an activator of protein kinase C (PKC) isoforms^5^. Notably, PLCɛ is unique in relation to other members of PLC family: it receives signaling inputs from both heterotrimeric G proteins and Ras/Rho small GTPases. Moreover, it is able to mediate sustained signaling via the generation of DAG in response to ligands that activate receptors coupled to Rho/Gα12/13^6^. Owing to its interaction with Ras family proteins, PLCɛ is generally deemed a tumor promoter. Indeed, a large number of studies have revealed the promoting role of PLCɛ in tumor development in a variety of cancer types^7–10^. Our previous study found that PLCɛ silencing reduced AR protein expression in CaP cells^11^. This urges us to explore whether PLCɛ is essential in preserving AR proteins in CaP.

Autophagy is a highly conserved process of protein degradation. Initially, autophagy was considered a process of nonspecific proteolysis to provide cells with energy or molecular materials when encountering stress conditions^12^. Recent studies have demonstrated that autophagy is also activated at basal levels and constantly programmed by numerous cellular signals, resulting in selective clearance of specific cargos^13^. Selective autophagy has now been reported to regulate the protein degradation of various molecules in cancer^14^.

In this study, we probed the relationship between PLCɛ and AR signaling in CaP. We demonstrated that PLCɛ could regulate AR protein degradation and AR nuclear translocation, both of which were achieved through AMPK/ULK1-mediated selective autophagy. Moreover, PLCɛ/AR signal axis was associated with the metastatic prowess of CaP and played a critical role in AR-antagonists resistance. Our data suggest that targeting this pathway is a potential treatment strategy for CaP and CRPC.

## Methods and materials

### Patient samples

Clinical samples were collected from patients in the Department of Urology of the First Affiliated Hospital of CQMU between July 2014 and January 2018. All samples were reevaluated by pathologists in CQMU (sample information see Supplementary STables 4 and 5). All patients provided informed consent. This study was approved by the Ethics and Research Committees of the First Affiliated Hospital of CQMU and conducted in accordance with the Declaration of Helsinki Principles.

### Immunohistochemistry

Immunohistochemical staining of samples was performed using a standard immunoperoxidase staining procedure (Antibody information see STable 6). A semi-quantitative scoring was used to count for both staining intensity and immunoreactivity ratio (STable 7). For statistical purposes, the samples grouped as negative/weak were considered stain-negative, while moderate or strong considered stain-positive.

### Cell culture

Cell culture information see STable 8. LNCaP-AD (Androgen-Deprivation) and LNCaP-CR (Castration-Resistant) were established by maintaining in RPMI-1640 with 10% charcoal stripped fetal bovine serum (Gibco, US) for 7 days or more than 3 months, respectively. To generate bicalutamide/enzalutamide-resistant cells, LNCaP cells were chronically exposed to increasing concentrations of bicalutamide/enzalutamide (1, 2, 5, 10, 20 μM). Cells that survive 2 μM bicalutamide/enzalutamide were designated as Bica-t/Enza-t. Cells that tolerate and maintain with 20 μM bicalutamide/enzalutamide for at least 4 months, were designated as Bica-R/Enza-R.

### Transfection

Ad-sh-NC and Ad-sh-PLCɛ were generated and transfected as previously described^11^. Subsequent assays or treatments (siRNA transfection, pharmaceuticals, etc.) were administered 48 h after transfection (siRNA and reagent information see STable 9 and 10). The siRNAs were mixed with 2.5 ml of INTERFERin (Polyplus-Transfection, France) in OPTI-MEM (Gibco, US), incubated at room temperature for 10 min and then added to cells. Medium was replaced and subsequent assays were conducted 36-48 h after transfection.

### Reverse transcription and real-time PCR

Total RNA was extracted using TRizol (Takara, Japan). Reverse transcription was performed using the Prime Script RT reagent kit (Takara, Japan) according to the manufacturer’s protocols. Real-time PCR was performed using the SYBR Premix Ex Taq™ II kit (Takara, Japan). The levels of mRNA were calculated using the comparative 2^−^^▵▵^^Ct^ method with β-actin serving as a calibrator. Primer information see STable 11. All gene expression experiments were performed at least five times.

### Western blot assay

Western blot assay was performed as previously described^11^. The intensity level of each protein band was quantified using Image-Pro plus 6.0. Antibody information see STable 12. Protein expression experiments were repeated three times.

### Immunofluorescence

Cells were cultured on sterile glass coverslips and subjected to different treatments. After indicated time, cells were fixed in 4% paraformaldehyde, treated with 0.1% Triton X-100, and blocked with 5% normal goat serum. Then, cells were ringed with PBS and incubated overnight at 4 °C in dilution buffer containing primary antibodies. After three times of washes with PBS, cells were incubated with an appropriate fluorochrome-conjugated secondary antibody for 1 h and subsequently stained with DAPI/4’,6-diamidino-2-phenylindole (Beyotime, China) for 15 min at 37 °C in the dark, followed by inspection using fluorescence microscopy (Keyence, Japan). Antibody information and experimental steps in detail see STable 13. Experiments were performed at least three times.

### Immunoprecipitation

Cells were harvested and washed twice with ice-cold PBS buffer. Immunoprecipitation was performed using the Pierce™ Co-Immunoprecipitation Kit (ThermoFisher, US) according to the manufacturer’s protocols. The inputs and the proteins bound to the resin were analyzed by western blot. Antibody information see STable 14. Experiments were performed at least three times.

### Reporter gene assay

Reporter plasmids pTATA-LUC, pPSA5.8-LUC^15^, pFKBP51(-3)-LUC (FKBP51-1)^16^, pFKBP51(12)-LUC (FKBP51-2)^17^ (Promega, US), and pCMVβ encoding β-galactosidase are purified from E. coli JM109 strain cultures using QIAGEN Plasmid Maxi Kit (Qiagen, Germany). Transfection of LNCaP and VCaP were carried out using jetPEITM transfection reagent according to manufacturer’s protocols (Polyplus-Transfection, France). Reporter gene assay was performed using TransDetect® luciferase reporter assay kit (TransGen, China) and Luciferase assay system (Promega, USA). The β-galactosidase-normalized LUC activities of each groups were documented. Experiments were performed at least three times.

### Wound healing assay

PLCɛ-silenced cells were seeded in 6 well plates and grown to 80% confluence. The “wound” was then created by scratching with a sterile 200 μL pipette and DHT (1 nM) or enzalutamide (20 μM) was added into the media. Images of wounds were captured at 0 and 24 h after scratching. Experiments were performed at least three times.

### Transwell assay

PLCɛ-silenced cells (1.5 × 10^4^ cells/well) were added to the upper well of the chamber, which contains a membrane filter (8 μm) and is inserted in 24-well plates (Millipore, US), and cultured with DHT (1 nM) or enzalutamide (20 μM) for 24 hours. For the invasion assay, diluted Matrigel (BD Biosciences, US) was used (2.5 × 10^4^ cells/well). Cells that pass through the membrane were stained with crystal violet and counted with a light microscope. Experiments were performed at least three times.

### CCK-8 assay

Cells were seeded in 96-well plates (2 × 10^3^ cells/well) for 12 h (day 0) and then subjected to various treatment. At the indicated time points, each well was added with 10 μL CCK-8 reagent solution (Beyotime, China), and incubated for 2 h. Optical density was determined using a microplate reader at the absorbance of 450 nm. Each group has five replicate wells and experiments were performed at least four times.

### Flow cytometry assay

PLCɛ-silenced Cells were seeded in 6 well plates, grown to 60% confluence, subjected to PLCɛ silencing for 48 h and finally treated with or without Enzalutamide (2 μM) for 24 h. Cells were collected and fixed with 75% ethanol. The cell cycle distribution was analyzed using flow cytometry. Experiments were performed at least three times.

### Colony formation assay

PLCɛ-silenced cells were seeded in 6 well plates (650 cells/well) and treated with Enzalutamide (2 μM). After 12 days of culture, adherent cells were washed twice with PBS, fixed with 4% paraformaldehyde (20 min) and stained with crystal violet solution (15 min). The colony number was counted using a light microscope. Colony-forming efficiency was calculated. (colony number/650) × %. Experiments were performed at least three times.

### Statistical analysis

All data were derived from at least three independent experiments (Four times for gene expression experiments, and five times for CCK-8 assay). Student’s *t*-test, Chi-square test, one-way ANOVA, Pearson’s correlation coefficient and Cohen’s kappa coefficient were used to evaluate the significant associations among categorical variables. The SPSS (Version 13.0) software and GraphPad (Prism 5) were used for statistical analyses. Data returning a value of *P* < 0.05 were considered statistically significant.

## Results

### PLCɛ expression is elevated in CaP tissues and associated with tumor metastasis

**Fig. 1:**
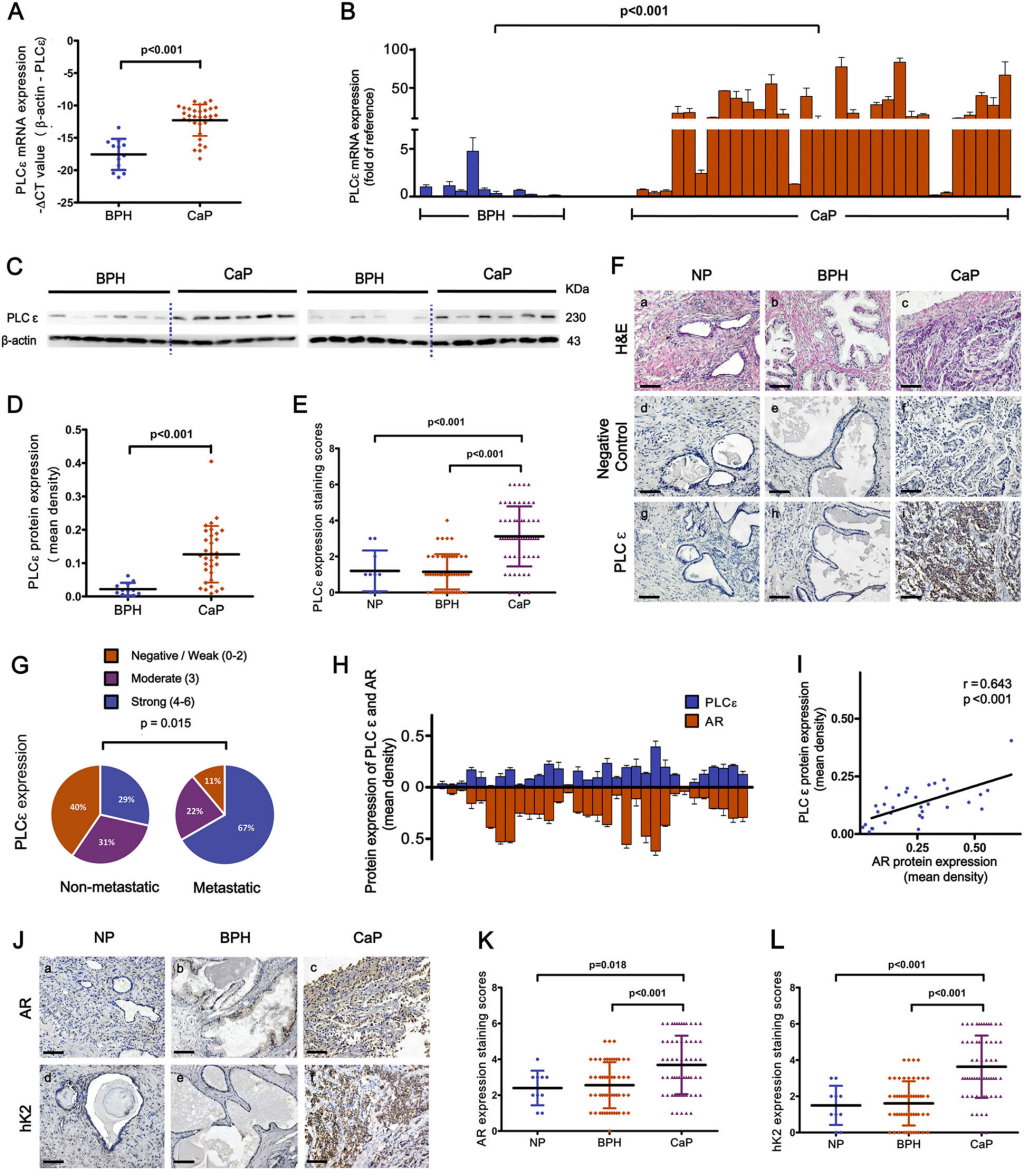
PLCɛ and AR signaling are closely related in CaP tissues. **a**–**d** PLCɛ expression in BPH and CaP samples. mRNA level of PLCɛ detected by RT-PCR was normalized to that of β-actin and shown in the form of -ΔCT value (**a**) or in a fold-change manner (specimen NO.1 as calibrator) (**b**). Protein expression was examined by western blot (**c**), and quantified as mean optical density (**d**). Average staining scores for PLCɛ expression in IHC analysis (**e**). Representative H&E and IHC staining: H&E (**a**–**c**). PBS served as negative control (**d**–**f**). PLCɛ (**g**–**i**). Scale bar, 100 μm (**f**). Differential PLCɛ expressions of metastatic CaP (*n* = 18) and non-metastatic CaP (*n* = 42) (**g**). Protein expressions of PLCɛ and AR in CaP samples (**h**). Correlation curve of PLCɛ protein versus AR protein in CaP samples (**i**). Representative IHC staining: AR (**a**–**c**). hK2 (**d**–**f**). Scale bar, 100 μm (**j**). Average staining scores for AR expression (**k**) and hK2 expression (**l**).

RT-PCR and western blot were performed on 32 CaP samples and 12 BPH samples to investigate PLCɛ expressions. The results showed that PLCɛ transcripts (Fig. 1a, b) and proteins (Fig. 1c, d and Supplementary Fig. 1) were significantly increased in CaP compared to BPH. Further validation was performed with immunohistochemical (IHC) analysis on a total of 60 CaP, 54 BPH and 10 NP samples. Semi-quantitative staining scores illustrated increased PLCɛ proteins in CaP compared to BPH and NP (Fig. 1e, f). The predominant cause of mortality from prostate cancer is metastasis, while PLCɛ could contribute to the metastatic prowess of some cancer types. Our data revealed that CaP samples from patients with metastatic loci had higher levels of PLCɛ protein than did those from non-metastatic patients (Fig. 1g).

### PLCɛ and AR signaling activity are closely related in CaP tissues

AR signaling is a crucial driver of CaP invasiveness. Therefore, AR proteins were further detected in 32 CaP samples through western blot (Fig. 1h and Supplementary Fig. 1). Pearson’s correlation coefficient analysis revealed a positive correlation between AR and corresponding PLCɛ in CaP (Fig. 1i). AR proteins were also explored using IHC in 60 CaP, 54 BPH and 10 NP samples (Fig. 1j). Staining scores showed that AR expression was up-regulated in CaP (Fig. 1k). Cohen’s kappa coefficient analysis indicated a substantial level of agreement between PLCɛ increase and AR increase (Kappa = 0.615, *P* < 0.001) (Table 1). Human kallikrein 2 (hK2) expression is under tight control of AR signaling, and it is deemed superior to AR, PSA and other markers when it comes to the evaluation of tissue AR signaling activity^2,4,18,19^. Our data showed that hK2 was also significantly increased in CaP (Fig. 1l) and tightly correlated with PLCɛ in CaP (Kappa = 0.668, *P* < 0.001) (Table 1). Taken together, these data indicate that higher level of PLCɛ can very likely be found in CaP samples exhibiting higher level of AR protein and, more importantly, higher level of AR signaling activity.

**Table 1:** Correlation between PLCɛ and AR/hK2 in CaP samples.

No. Specimens	PLCɛ			
AR	Negative/Weak	Moderate	Strong	Total
Negative/Weak (0–2)	14	2	1	17
Moderate (3)	3	9	1	13
Strong (4–6)	2	6	22	30
Total	19	17	24	60
	Kappa value = 0.615		*P* value < 0.001	

hK2	Negative/Weak	Moderate	Strong	Total
Negative/Weak (0–2)	15	1	0	16
Moderate (3)	4	10	2	16
Strong (4–6)	0	6	22	28
Total	19	17	24	60
	Kappa value = 0.668		*P* value < 0.001	

*P* values were calculated using the Cohen’s kappa coefficient.

### PLCɛ depletion blocks AR signaling in CaP cell lines

**Fig. 2:**
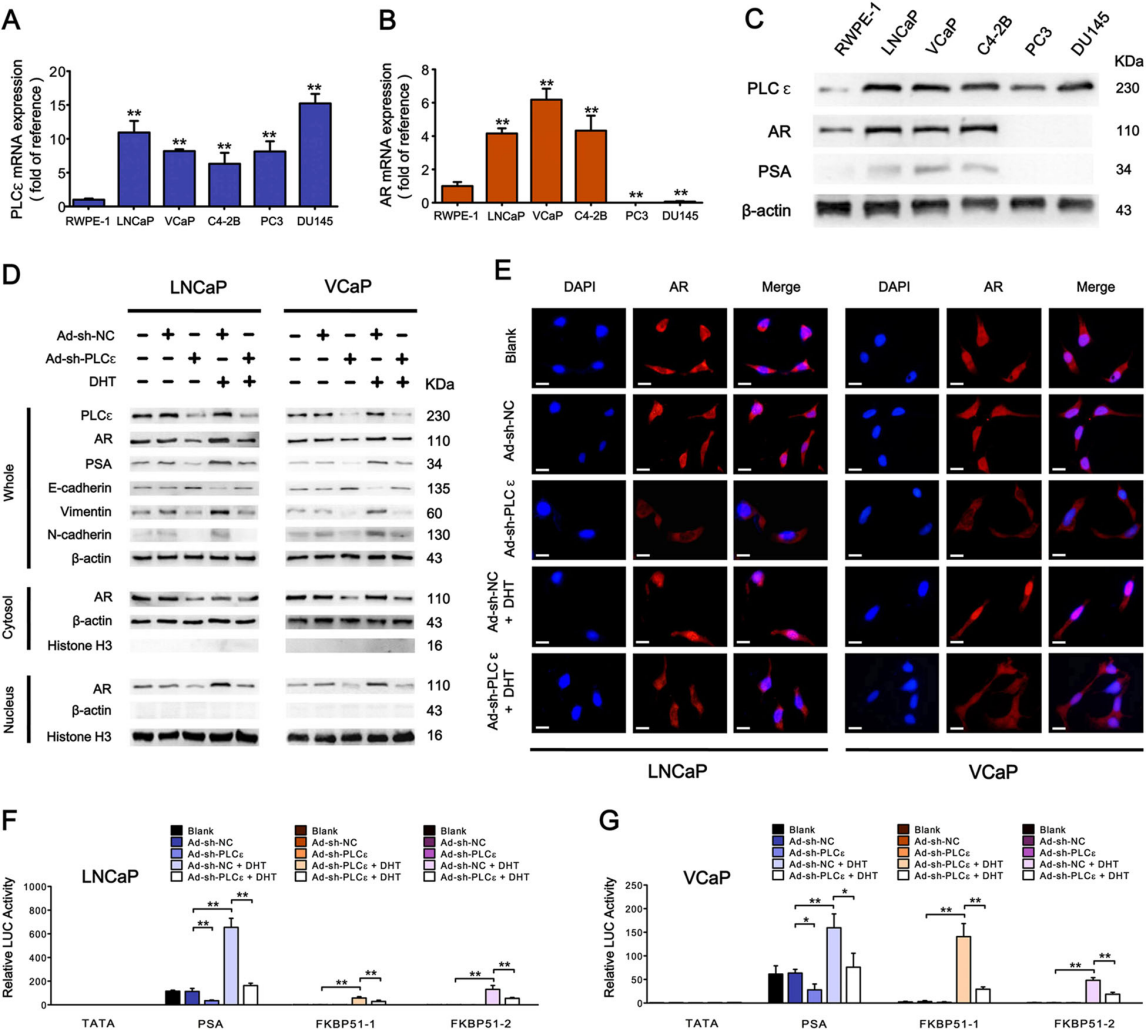
PLCɛ depletion blocks AR signaling in CaP cell lines. **a**–**c** PLCɛ and AR expressions in different prostate cell lines: RT-PCR illustrates mRNA levels of PLCɛ (**a**) and AR (**b**) (“RWPE-1” as calibrator; * compared to “RWPE-1”). Western blot illustrates protein levels of PLCɛ, AR, and PSA (**c**). Protein expressions of PLCɛ, AR, and other molecules in LNCaP/VCaP under PLCɛ depletion with or without further treatment of DHT (1 nM) for 12 h (**d**). Immunofluorescence staining analysis of AR intracellular distribution, Scale bars, 10 μm (**e**). Transcriptional activity of AR assessed by reporter gene assays: LNCaP (**f**) or VCaP (**g**) were transfected with pTATA-LUC, pPSA5.8-LUC or LUCs driven by two different FKBP51 enhancer fragments, and then subjected to PLCɛ depletion with or without treatment of DHT (1 nM) before reporter analysis. Data are shown as relative LUC activity. Significance: *p < 0.05, **p < 0.01.

Figure 2a–c show that PLCɛ transcripts and proteins were remarkably increased in all five CaP cell lines compared to RWPE-1, a normal prostate epithelial cell line. AR and PSA were significantly elevated in LNCaP, VCaP, and C4-2B, but completely lost in PC3 and DU145. PLCɛ expression was then silenced using the most effective targeting-shRNA as described in our previous researches^9,11^ in LNCaP and VCaP. The results showed that PLCɛ knockdown reduced AR expression with or without DHT treatment (Fig. 2d). Furthermore, AR nuclear translocation was inhibited after PLCɛ depletion even under ligand stimulation (Fig. 2d, e). PSA, which reflects AR signaling activity, was also down-regulated after PLCɛ knockdown (Fig. 2d). To directly evaluate the alteration of AR signaling, we performed reporter gene assays. A PSA promoter- and two FKBP51 enhancer-driven firefly luciferase (LUC) gene reporters were chosen for the measurement of AR transcriptional activity. The data revealed that PLCɛ depletion inhibited PSA promoter activity, and moreover reversed the increased activity of three different AR-regulated LUC reporters prompted by DHT (Fig. 2f, g). Taken together, these data suggest that the effect of PLCɛ depletion predominates over that of ligand stimulation, thereby strongly blocking AR signaling activation.

### PLCɛ is essential in AR-signaling-driven cell migration and invasion

**Fig. 3:**
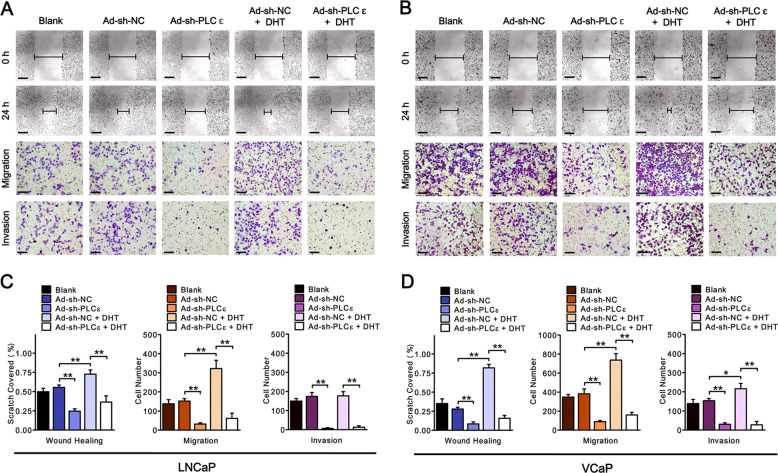
PLCɛ is indispensable in AR-driven cell migration/invasion. Wound healing assay shows the coverage of the scratched area at 0 or 24 h after DHT stimulation in LNCaP. Scale bars, 100 μm (**a**). Transwell migration/invasion assays were carried out when DHT was added and the incubation time was 24 h. Scale bars, 40 μm (**a**). Statistical analysis (**c**) Similar data obtained from VCaP (**b**). Statistical analysis (**d**). Significance: *p < 0.05, **p < 0.01.

E-cadherin, Vimentin and N-cadherin were measured to evaluate the metastatic (migration/invasion) shift in marker expression after PLCɛ depletion. As shown in Fig. 2d, Vimentin and N-cadherin were elevated under DHT stimulation, but greatly impaired after PLCɛ knockdown. However, E-cadherin was just the opposite. Wound healing and transwell assays (with or without Matrigel coating) in LNCaP (Fig. 3a, c) and VCaP (Fig. 3b, d) further showed that PLCɛ depletion attenuated cell migration and invasion in the absence and presence of DHT treatment, which was in line with the above-mentioned metastatic markers. All in all, these results demonstrate the essential role of PLCɛ in AR-signaling-mediated cell migration and invasion.

### PLCɛ regulates AR degradation via autophagic pathways

**Fig. 4:**
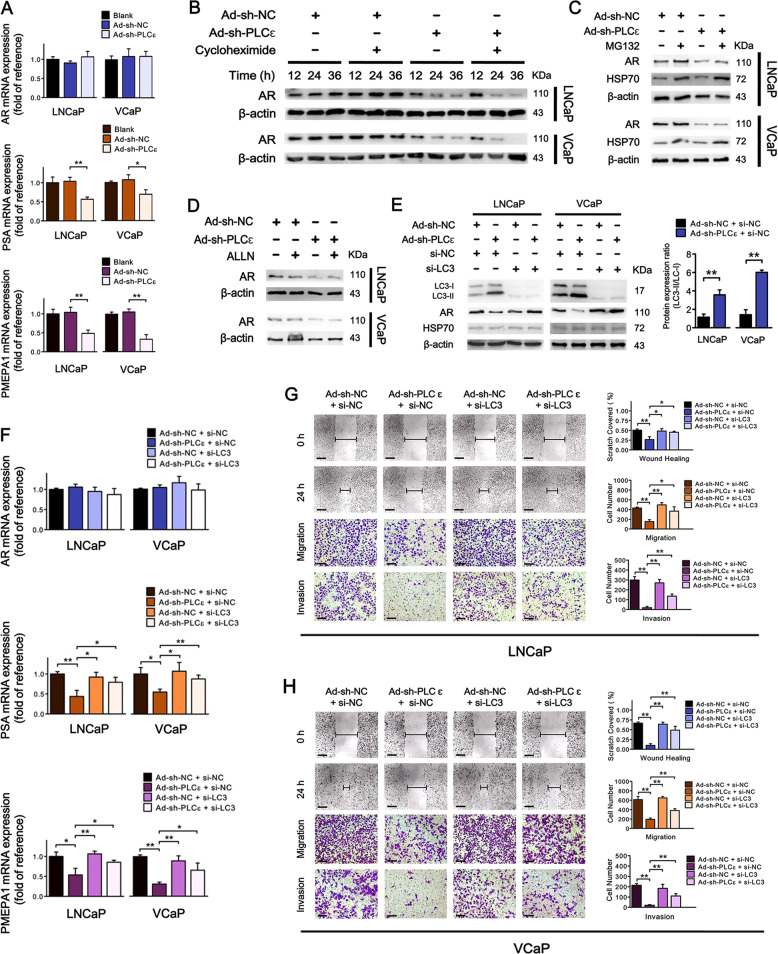
PLCɛ regulates AR degradation via autophagic pathways. RT-PCR analysis of AR, PSA and PMEPA1 mRNA in LNCaP/VCaP after PLCɛ silencing (**a**). Time-course analysis of AR protein in cells subjected to PLCɛ silencing with or without pretreatment with cycloheximide (30 μM) for 4 h (**b**). Protein expressions of AR and HSP70 in cells subjected to PLCɛ depletion, followed by culturing with or without MG132 (10 μM) for 18 h (**c**). AR protein in cells subjected to PLCɛ depletion in the absence or presence of ALLN (30 μM) (**d**). Protein expressions of LC3-I/LC3-II, AR, and HSP70 in cells subjected to PLCɛ depletion and LC3 silencing (si-LC3 #1), alone or combined (**e**). RT-PCR analysis of AR, PSA, and PMEPA1 mRNA levels (**f**). Wound healing assay (Scale bars, 100 μm) and transwell assays (Scale bars, 40 μm) were carried out in LNCaP after the completion of si-NC/ si-LC3 transfection. The incubation time for transwell assays was 24 h (**g**). Similar data obtained from VCaP (**h**). Significance: *p < 0.05, **p < 0.01.

We next probed how PLCɛ regulates AR expression. As shown in Fig. 4a, PLCɛ knockdown only down-regulated mRNA levels of PSA and PMEPA1 (androgen-induced genes), but not AR itself. In addition, in cycloheximide-treated cells, PLCɛ knockdown caused a more significant decrease of AR protein in a time-dependent manner (Fig. 4b). These results suggest that the reduced AR level in this event could be largely attributed to protein degradation instead of synthesis. Ubiquitin-proteasome degradation is a common method for AR proteolysis in CaP^20^, whereas calpain-mediated AR degradation under control of intracellular Ca^2+^ level is also reported to be less common in prostate cancer^21^. However, blocking neither ubiquitin-proteasome degradation pathway (MG132) (Fig. 4c) or calpain-mediated degradation pathway (ALLN) (Fig. 4d) could not reverse the effect of PLCɛ depletion on AR expression. Instead, PLCɛ knockdown induced a conversion of LC3-I to LC3-II (Fig. 4e), suggesting a possible alternative of protein degradation associated with enhanced autophagy. Impairment of autophagic pathway via LC3 depletion (si-LC3 #1) neutralized the effect of PLCɛ knockdown on AR expression and its targeting gene transcriptions (Fig. 4e, f), and restored cell migration/invasion in PLCɛ knockdown groups (Fig. 4g, h). These results were further validated using another two small interfering RNAs (si-LC3 #2, #3) in VCaP cells (Supplementary Fig. 2). Together, these data suggest that AR degradation after PLCɛ knockdown is highly associated with autophagic pathway.

### PLCɛ depletion triggers enhanced autophagic activity

**Fig. 5:**
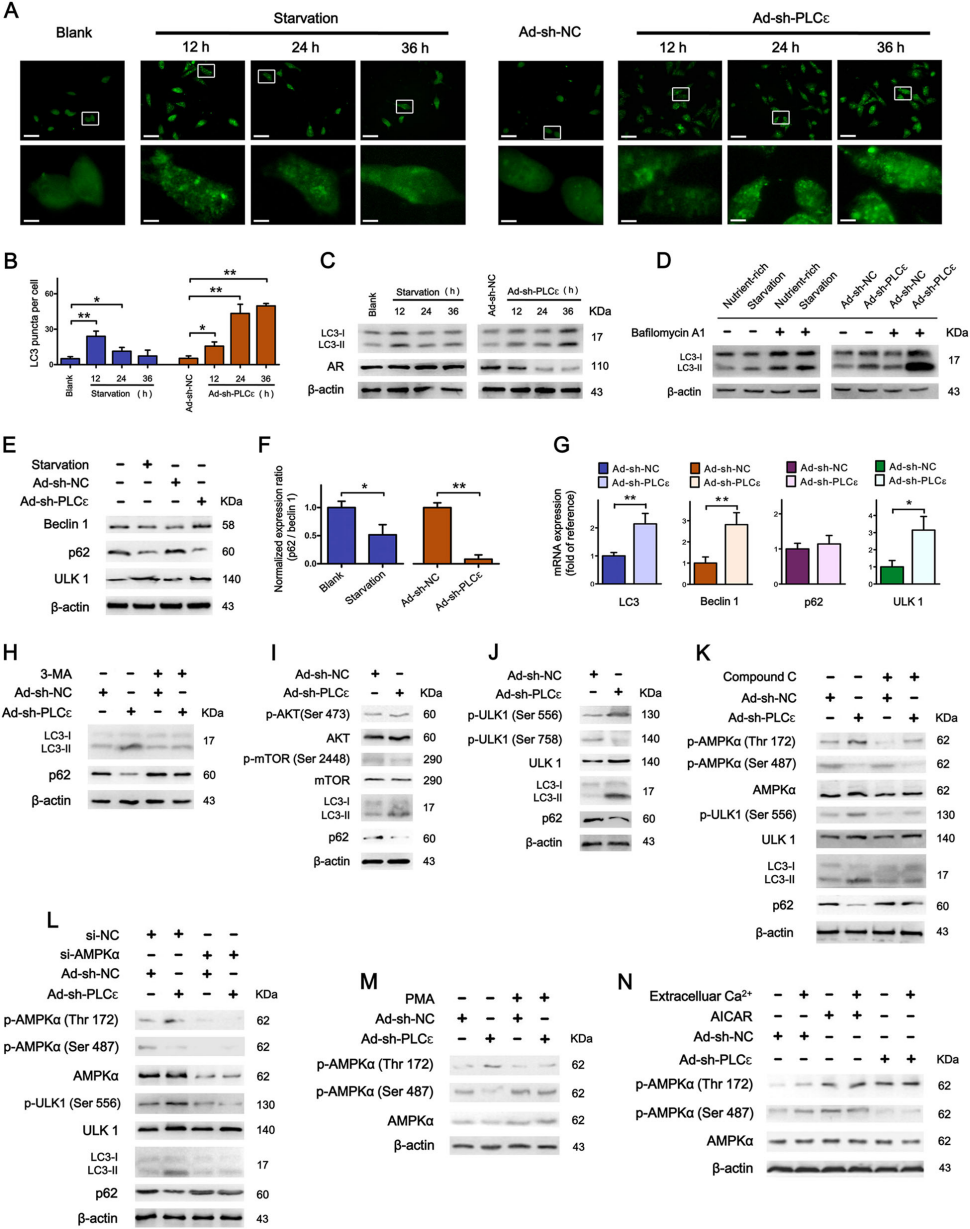
PLCɛ depletion causes the induction of autophagy. **a**–**c** Cells were cultured in full medium (RPMI-1640 plus 10% FBS for group “Blank”, “Ad-sh-NC” and “Ad-sh-PLCɛ“) or starvation medium (50% RPMI-1640 plus 50% EBSS for group “Starvation”): LC3 puncta (LC3-II accumulation) were visualized by immunofluorescence. Scale bars, 30/4 μm (**a**). Quantification of LC3 puncta at indicated time (**b**). Protein expressions of LC3-I/LC3-II and AR (**c**). LC3-I/LC3-II expression in LNCaP subjected to PLCɛ silencing with or without subsequent treatment of bafilomycin A1 (100 nM) for 24 h (Starvation group served as positive control (**d**). Protein expressions of beclin 1, p62 and ULK 1 in LNCaP treated with starvation for 24 h or PLCɛ silencing for 48 h (**e**, **f**). RT-PCR analysis of LC3, beclin 1, p62, and ULK 1 mRNA in LNCaP subjected to PLCɛ silencing (**g**). Effect of 3-MA (3 mM for 8 h) on autophagic flux (**h**). Phosphorylation levels of AKT, mTOR and ULK1 in LNCaP subjected to PLCɛ silencing (**i**, **j**). Effect of compound C (3 μM for 4 h) on AMPK activation, ULK1 phosphorylation and autophagic flux (**k**). Effect of AMPKα silencing (**l**). Effect of PMA (5 μM for 2 h) on AMPK activation (**m**). Effect of Ca^2+^ concentration on AMPK activation: PLCɛ-silenced LNCaP were incubated in the presence or absence of extracellular Ca^2+^ for 2 h with AICAR stimulation (1 mM, 2 h) serving as control (**n**). Significance: *p < 0.05, **p < 0.01.

Immunofluorescence showed that PLCɛ knockdown induced an increased number of LC3 puncta (accumulation) in LNCaP, and it reached the apex at 36 h. Starvation groups served as positive control (Fig. 5a, b). Western blot correspondingly revealed higher LC3-II/LC3-1 ratio in groups with more LC3 puncta (Fig. 5c and Supplementary Fig. 3A). The accumulation of LC3 can be the result of either an enhanced formation or a reduced degradation of autophagosomes, indicative of an increased or decreased autophagic activity respectively^12^. We observed a supra-additive effect of LC3-II level in cells simultaneously treated with PLCɛ silencing and bafilomycin A1 (autophagosome-lysosome inhibition), which suggests an enhanced formation of autophagosomes after PLCɛ silencing (Fig. 5d and Supplementary Fig. 3B). Moreover, p62 was significantly down-regulated after PLCɛ depletion, whereas the levels of beclin 1 and ULK 1 were elevated (Fig. 5e). A dramatic decline was observed in the ratio of p62 to beclin 1 in PLCɛ knockdown group (Fig. 5f), which is primarily observed in an enhanced autophagic flux^13^. The transcripts of LC3, beclin 1 and ULK 1 were also increased after PLCɛ depletion, suggesting cells undergoing prolonged elevation of autophagic activity (Fig. 5g). This demonstrates that PLCɛ knockdown can trigger sustained and elevated autophagic activities.

### PLCɛ depletion causes the onset of autophagy via AMPK/ULK1 pathway

Autophagy is a multistep biological process and PLCɛ may regulate it by targeting its onset or/and other steps. 3-MA is a sequestration inhibitor, which prevents autophagy initiation and it strongly abrogated PLCɛ-depletion-mediated autophagy enhancement (Fig. 5h and Supplementary Fig. 3C), implying that PLCɛ depletion functions as an autophagic initiator. Moreover, PLCɛ depletion did not influence PI3K/AKT/mTOR pathway, which is classically related to autophagy initiation (Fig. 5i and Supplementary Fig. S3D). Instead, it caused phosphorylation of ULK 1 at serine 556, a AMPK site that induces autophagy, and dephosphorylation of ULK 1 at serine 758, a site that inhibits autophagy by preventing the interaction between ULK1 and AMPK (Fig. 5j and Supplementary Fig. 3E)^22^. Further study showed that PLCɛ knockdown could activate AMPK, evidenced by phosphorylation of AMPKα at threonine 172 and dephosphorylation at serine 487 (Fig. 5k)^23^. AMPK inhibitor compound C (Fig. 5k and Supplementary Fig. 3f) and AMPKα depletion (Fig. 5l and Supplementary Fig. 3g) could greatly attenuate the enhanced autophagy caused by PLCɛ knockdown, verifying the participation of AMPK activation in autophagy initiation. PLCɛ performs biological functions mainly through activating PKC enzymes and subsequently regulating the intracellular Ca^2+^ level^6^. By using PMA, a strong activator of PKC pathway, we discovered that PKC inhibition was essential for AMPK activation after PLCɛ knockdown (Fig. 5m). However, the deprivation of extracellular Ca^2+^ failed to affect AMPK phosphorylation induced by either PLCɛ knockdown or AICAR stimulation, a non-Ca^2+^-dependent AMPK activator serving as positive control (Fig. 5n). This suggests that PLCɛ/PKC-mediated Ca^2+^ alteration may not be the predominant determinant in AMPK activation.

### PLCɛ depletion induces selective autophagy and promotes formation of p62-AR and NBR1-AR complexes in cytosol

**Fig. 6:**
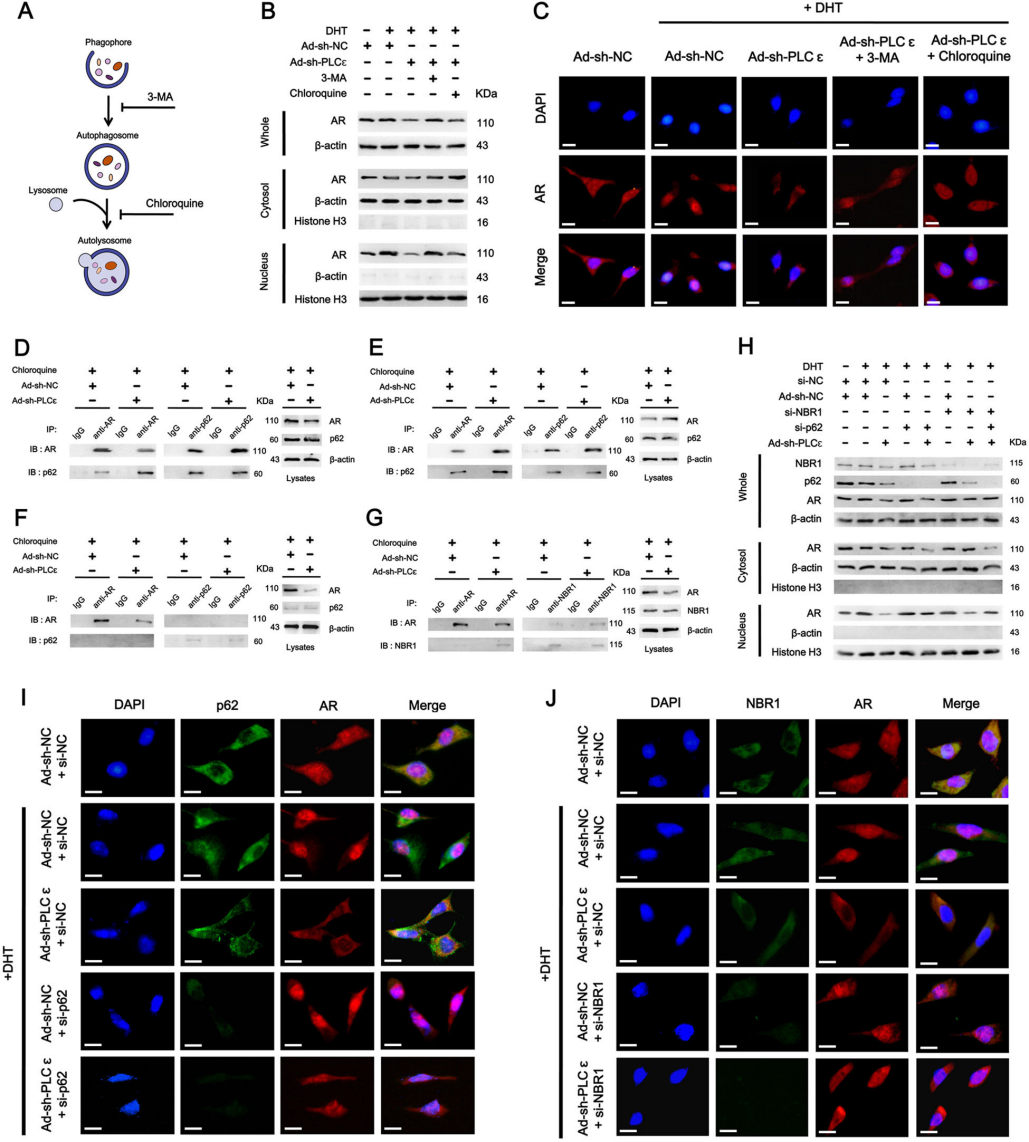
PLCɛ depletion promotes the formation of p62-AR and NBR1-AR complexes in cytosol. The mechanism of action of autophagy inhibitors (**a**). AR protein in cells under PLCɛ silencing with or without further treatment of 3-MA (3 mM) or chloroquine (50 μM) for 12 h (**b**). Immunofluorescence staining showing AR intracellular distribution. Scale bars, 10 μm (**c**). **d**–**g** The formation of p62-AR and NBR1-AR complexes: PLCɛ-silenced cells were subjected to chloroquine (25 μM) for 2 h. Total lysates were immunoprecipitated (IP) with anti-AR, anti-p62 or control IgG antibodies, followed by immunoblotting (IB) for AR and p62, respectively (**d**). Immunoprecipitation assay of cytosolic lysates (**e**) and nuclear lysates (**f**). Total lysates were “IP” with anti-AR, anti-NBR1 or control IgG antibodies, followed by “IB” for AR and NBR1 (**g**). **h**–**j**: PLCɛ-silenced cells were subjected to p62 and NBR1 silencing, alone or combined: Protein expressions of AR, p62, and NBR1 (**h**). Intracellular localizations of AR and p62 visualized by immunofluorescence. Scale bars, 10 μm (**i**). Intracellular localizations of AR and NBR1. Scale bars, 10 μm (**j**).

The inhibitors of autophagy function through highly different mechanisms (Fig. 6a). Both 3-MA, an initiation inhibitor, and chloroquine, a lysosomal inhibitor, could restore total AR expression after PLCɛ knockdown, with 3-MA exhibiting a stronger effect (Fig. 6b). In addition, 3-MA restored AR primarily in nucleus, whereas chloroquine primarily in cytosol (Fig. 6b, c), implying that PLCɛ could also play a role in formation of AR-containing autophagosomes. Furthermore, PLCɛ depletion induced the phosphorylation of autophagy adaptor p62 (serine 403) (Supplementary Fig. 4), indicative of selective autophagy.

The widely accepted conception that stress-induced “bulk” autophagy is unselective whereas specific pathway-regulated or adaptor-driven basal autophagy is selective, is also in favor of PLCɛ-mediated AR removal resulting from the selective autophagy^24,25^. Immunoprecipitation assay revealed that AR antibody pulled down AR proteins along with a significantly higher level of p62 in PLCɛ knockdown group. Similarly, p62 antibody also pulled down a higher level of p62-AR complex in PLCɛ knockdown group, implicating an enhanced interaction between AR and p62 (Fig. 6d). Subcellular localization analysis showed that PLCɛ knockdown triggered increased p62-AR interactions in the cytosol (Fig. 6e), but not in the nucleus (Fig. 6f). In addition, PLCɛ depletion also slightly enhanced interaction between AR and NBR1, an autophagy adaptor which is complementary to p62 in mediating selective autophagy (Fig. 6g). This was verified by our data showing that only silencing both p62 and NBR1 could fully restore AR after PLCɛ depletion (Fig. 6h). Interestingly, silencing either p62 or NBR1 was capable of reversing PLCɛ-depletion-induced AR nuclear translocation to varying degrees. Immunofluorescence revealed that AR and p62 were co-localized in cytosol after PLCɛ depletion, whereas p62 silencing resulted in AR localizing to nucleus (Fig. 6i). NBR1 depletion caused similar changes of intracellular AR distribution (Fig. 6j). Taken together, PLCɛ knockdown could promote adaptor-driven autophagosome formation and give rise to “anchoring effect” (see Discussion), resulting in AR degradation and nuclear translocation inhibition, respectively.

### PLCɛ and AR are simultaneously increased in AR-antagonist-resistant CaP cell lines

**Fig. 7:**
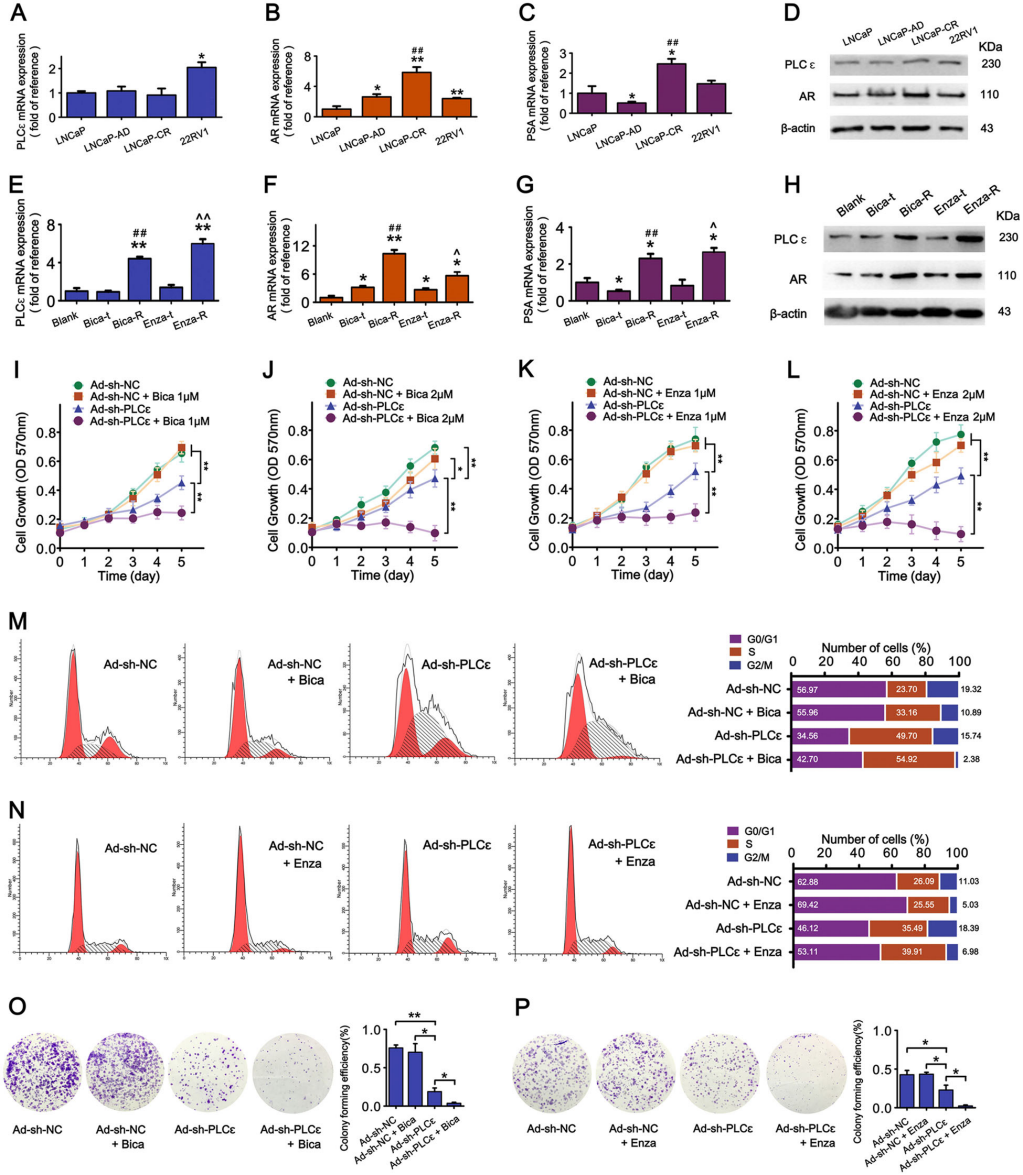
PLCɛ/AR pathway is involved in CaP resistance against AR antagonists. **a**–**d** The mRNA and protein expressions of PLCɛ, AR and PSA. (“LNCaP” as calibrator; * compared to “LNCaP”; # compared to “LNCaP-AD”). **e–h** The mRNA and protein expressions of PLCɛ, AR and PSA. (“Blank” as calibrator; * compared to “Blank”; # compared to “Bica-t”; ^ compared to “Enza-t”). **i–l** Cell viability measured by CCK-8 assay every 24 h for 5 days. **m–p** Cell cycle phase distribution (**m**, **n**) or colony-forming efficiencies (**o**, **p**) of LNCaP subjected to PLCɛ-depletion or/and bicalutamide/enzalutamide (2 μM). Significance: *p < 0.05, **p < 0.01, ^*p* < 0.05, ^^*p* < 0.01, ##*p* < 0.01.

Persistent activation of AR signaling is a major impetus of CRPC^4^. Therefore, we hypothesized that high level of PLCɛ maintains AR overactivation and subsequently contributes to resistance against ADT. ADT is generally performed via castration or/and AR antagonist (anti-androgen), and accordingly we established two groups of LNCaP derivatives representing two types of resistances. In castration-resistant groups (LNCaP-AD, LNCaP-CR and 22RV1 as control), although AR were gradually increased, PLCɛ remained unchanged (Fig. 7a–d), suggesting PLCɛ may not play a role. However, in AR-antagonist-resistant groups, Bica-R/Enza-R had both increased PLCɛ and AR (Fig. 7e–h). Moreover, the transitional Bica-t/Enza-t also exhibited molecular changes that align very well with our hypothesis (see Discussion). This suggests an association between PLCɛ and AR in resistance against AR antagonist, but not simple castration.

### PLCɛ is indispensible during CaP resistance against AR antagonists

CCK-8 showed that initial concentration (1 μM) of bicalutamide barely affected wild-type cells, but substantially inhibited viability of PLCɛ-silenced cells (Fig. 7i). In addition, when the concentration increased (2 μM), PLCɛ depletion and bicalutamide had a much stronger synergistic effect, rendering it impossible for PLCɛ-silenced cells to survive chronically escalating bicalutamide concentrations during Bica-R establishment (Fig. 7j). Similar data were obtained using enzalutamide (Fig. 7k, l). Flow cytometry showed that bicalutamide/enzalutamide and PLCɛ depletion synergistically inhibited cell proliferation through S phase arrest (Fig. 7m, n), indicating cell viability inhibition resulted from decreased cell proliferation instead of drug toxicity. In colony formation assay, almost no standard colony were formed in group of bicalutamide/enzalutamide plus PLCɛ-silencing (Fig. 7o, p). These results suggest the essential role of PLCɛ in cell growth during AR-antagonist resistance.

### PLCɛ/AR axis is associated with the metastatic prowess of Bica-R-CaP

**Fig. 8:**
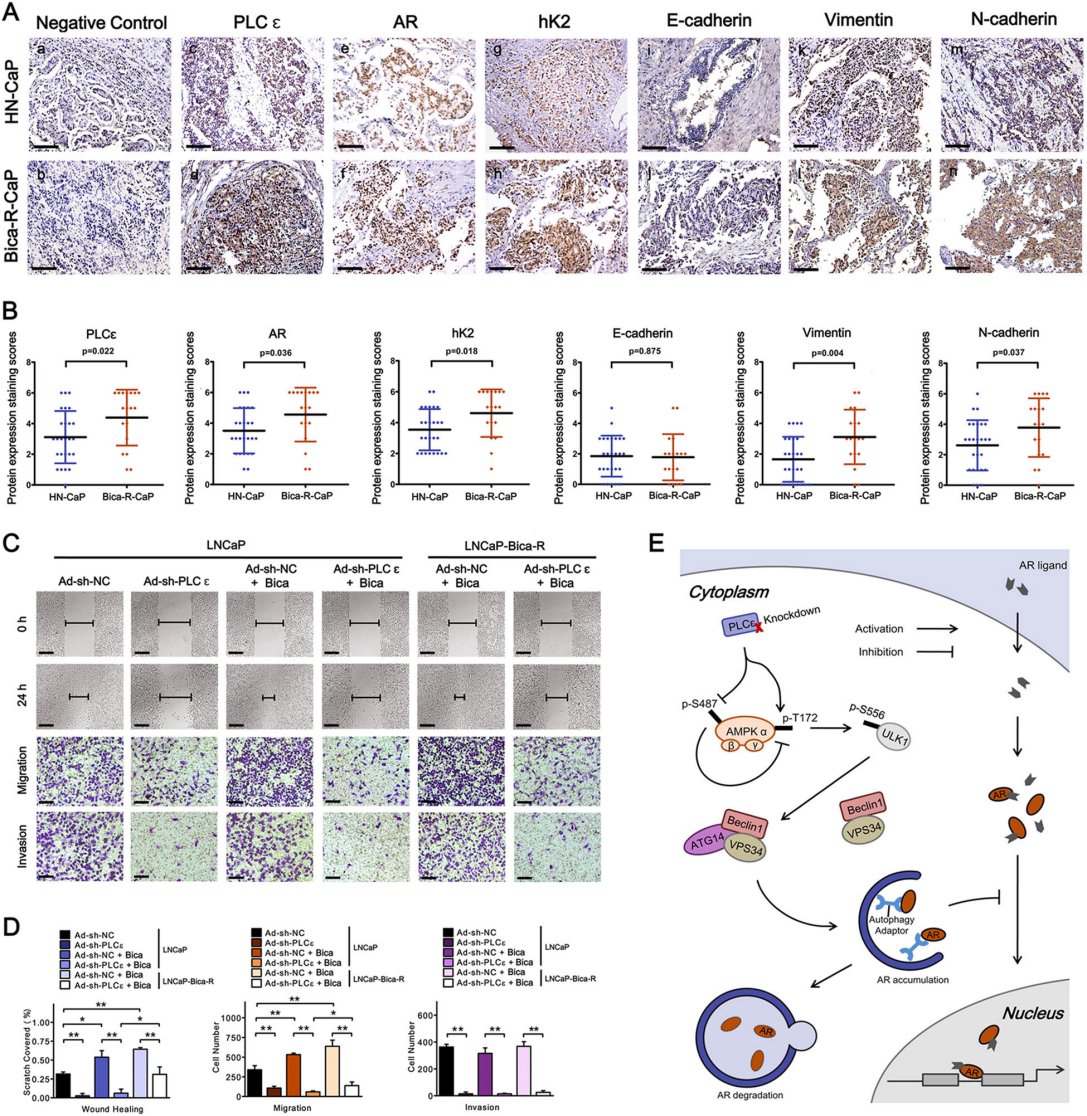
PLCɛ/AR axis is associated with the metastatic prowess of Bica-R-CaP. Representative IHC staining: PBS served as negative control (**a**, **b**). PLCɛ (**c**, **d**). AR (**e**, **f**). hK2 (**g**, **h**). E-cadherin (**i**, **j**). Vimentin (**k**, **l**). N-cadherin (**m**, **n**). Scale bars, 100 μm (**a**). Average staining scores in IHC analysis (**b**). Migration/invasion of LNCaP and LNCaP-Bica-R subjected to PLCɛ silencing and bicalutamide treatment (20 μM), alone or combined: Wound healing assay (Scale bars, 100 μm) and transwell migration/invasion assays (scale bars, 40 μm) were carried out as above-mentioned (**c**). Statistical analysis (**d**). Schematic diagram describing the molecular mechanism of AR signaling inhibition after PLCɛ knockdown (**e**).

We next examined PLCɛ/AR axis and CaP metastatic property in samples from patients receiving no ADT (HN-CaP) and patients who were bicalutamide-resistant (Bica-R-CaP), respectively. Data from immunohistochemical analysis upon 18 Bica-R-CaP and 26 HN-CaP samples showed that PLCɛ, AR, hK2, Vimentin and N-cadherin were increased in Bica-R-CaP compared to HN-CaP (Fig. 8a, b). No difference was observed in E-cadherin levels. Analysis of Cohen’s kappa coefficient again indicated that PLCɛ increase and AR increase had a substantial level of agreement in HN-CaP (kappa = 0.570, *p* = 0.003) and even higher in Bica-R-CaP (kappa = 0.824, *P* < 0.001). PLCɛ increase was also associated with hK2 increase in both HN-CaP (kappa = 0.662, *p* < 0.001) and Bica-R-CaP (kappa = 0.609, *P* = 0.005) (Supplementary Table 1). Supplementary Tables 2 and 3 show that Vimentin and N-cadherin were associated with PLCɛ and AR signaling in both sample types (except for Vimentin/hK2 increase). E-cadherin decrease was in moderate consistency with PLCɛ increase, but not with AR signaling. Our in vitro study revealed LNCaP-Bica-R exhibited higher migration ability than wild-type LNCaP. PLCɛ depletion could markedly suppress LNCaP-Bica-R migration, but not as effective as in wild-type LNCaP. The invasion abilities of LNCaP-Bica-R and wild-type LNCaP cultured with or without bicalutamide did not differ significantly, but they were all inhibited by PLCɛ-depletion (Fig. 8c, d). These results suggest that PLCɛ/AR pathway is also tightly associated with the metastatic property of bicalutamide-resistant CaP.

## Discussion

The term “oncogene addiction” describes a phenomenon that a single gene functions as the persistent driver of a certain malignancy^26^, and this concept perfectly portrays the relationship between AR signaling and CaP. Recent studies have revealed that AR signaling plays a critical role throughout all the stages of CaP, including carcinogenesis, primary cancer, CRPC and even CRPC resistant to AR antagonists^4^. This addiction indicates that certain ‘fail-safe’ mechanisms must have been established in CaP in order to sustain a functional AR signaling. Because of the importance of AR pathway in normal cellular physiology, an intrinsic capability of cells to maintain AR signaling is already existent, particularly in males who only have one single copy of AR gene and in prostate which is highly dependent on the AR axis to function properly. These biological mechanisms could be readily manipulated and pathologically strengthened by CaP cells to over-activate AR pathway. In this study, we disclosed a new mechanism underlying persistent AR signaling in CaP: Increased PLCɛ level inhibits AR protein degradation and facilitates AR nuclear translocation, resulting in enhanced AR signaling activity and cellular transformation toward a pro-metastatic phenotype.

AR pathway is frequently implicated in CaP tumorigenesis and metastasis^3^. Similarly, high PLCɛ level is reported to facilitate tumor development and metastasis in a number of cancer types as well^7–9^. In the present work, we found that increased PLCɛ expression was also a key contributor to the metastatic prowess of CaP. Moreover, PLCɛ expression and AR signaling were closely associated in CaP samples. Certain EMT phenotypes such as impaired E-cadherin expression or aberrant induction of Vimentin and N-cadherin are related with enhanced tumor metastasis^27^. Our data from clinical samples revealed good correlations among PLCɛ protein, AR signaling, Vimentin and N-cadherin in both hormone-naive CaP tissues and CRPC tissues. These results were further verified in cell study. We discovered that PLCɛ depletion was sufficient to suppress AR pathway and AR-driven cell migration/invasion in different CaP cell lines. PLCɛ could induce the alterations of E-cadherin, Vimentin and N-cadherin toward a phenotype favoring tumor metastasis. These lines of evidence strongly support the indispensable role of PLCɛ in promoting AR pathway activity and AR-mediated CaP metastasis. Our data from AR-antagonist resistant CaP also support PLCɛ sustaining AR signaling. We found that Bica-t/Enza-t had almost unaltered PLCɛ and AR protein level, even the synthesis of AR protein seemed to be up-regulated (evidenced by increased mRNA level). Based on our data that PLCɛ protects AR protein from degradation, it is rational to hypothesized that, without concomitant high PLCɛ level, the net protein of AR is unlikely to be elevated accordingly. Whereas in fully bicalutamide/enzalutamide-resistant cells, PLCɛ and AR were simultaneously increased. Constant up-regulation of AR protein is the impetus of CRPC. Therefore, PLCɛ elevation could be playing both a triggering role and a sustaining role in AR-antagonist resistance.

Autophagy is an important way of protein degradation. Studies of recent years have been highlighting the function of selective autophagy, which induces selective clearance of specific cargos. In that case, autophagy becomes a part of the intricate networks of cellular signals instead of simply a process in reaction to stress, and its effect is largely dependent on the specific proteins that are eliminated^13^. AR protein has been reported to be the cargos of autophagic degradation^28–31^. In our study, AR protein was also found to be degraded via an autophagy-dependent pathway when PLCɛ was silenced. Moreover, our data showing that PLCɛ depletion initiated p62-mediated AR degradation support the involvement of selective autophagy, because it is generally recognized that selective autophagy is regulated by autophagy adaptors/receptors, such as p62, NBR1, OPTN, NDP52, etc. Recent studies illustrate that p62 can sometimes be degraded through non-selective autophagy as well^24^. Therefore, we further investigated another autophagy receptor-- NBR1. The results revealed that p62 and NBR1 could function cooperatively in the regulation of AR degradation, providing one more evidence for selective autophagy. Notably, we observed that PLCɛ depletion induced the formation and accumulation of p62-AR and NBR1-AR complex in the cytoplasm. This is in accordance with previous studies reporting that p62 and ubiquitinated proteins often exert an anchoring effect on each other when the intracellular ubiquitin level is increased, which in turn causes the decreased cytoplasmic/nuclear shuttling of p62 and targeted proteins^29,32^. Therefore, owing to the cytosolic accumulation of AR complexes, PLCɛ depletion could dampen AR signaling via inhibition of nuclear translocation, even when the lysosomal degradation is blocked or lagged. This suggests that PLCɛ-mediated suppression of autophagic activity, in addition to its role in maintaining a high level of the AR protein, could facilitate AR translocation by preventing the anchoring effect, consequently preserving the functionality of AR signaling in CaP cells.

In literature, ‘PLC-related’ molecules have been implicated as autophagy inhibitors, such as IP_3_, IP_3_ receptor and PKC^33,34^. These reports are consistent with our data that PLCɛ could suppress autophagy by activating PKC pathway. Furthermore, we demonstrated that AMPK activation is essential for the enhancement of autophagic activity after PLCɛ depletion. Although PKC pathway and AMPK pathway are commonly considered calcium-regulated, we found that PLCɛ depletion could regulate AMPK pathway in a PKC-dependent but calcium-independent manner. Previous studies have shown that PKC is able to reduce AMPK activity by phosphorylating serine 487, a site that blocks threonine 172 phosphorylation and functions as a negative-feedback regulation of AMPK activation^23,35^. Therefore, it is logical to infer that PLCɛ depletion could impair the phosphorylation of serine 487 in AMPK via PKC inhibition, resulting in threonine 172 phosphorylation and subsequent AMPK activation due to decreased negative feedback suppression. This could happen while not needing a dramatic change of intracellular Ca^2+^ level. This mechanism is also in line with the hypothesis that PLCɛ is associated with basal autophagy. Because PLCɛ is the only member of PLC isoenzymes that is capable of triggering sustained and steady cellular signal change, while other members always mediate sudden and intensive calcium flow, which tend to induce endoplasmic reticulum stress, a process that is highly associated with non-selective autophagy.

Collectively, our study demonstrates that increased PLCɛ in CaP inhibits basal autophagic activity to a certain level of equilibrium, which is sufficient to maintain a high level of AR function, hence the increased metastatic prowess and drug resistance in CaP. Disruption of this equilibrium can result in weakened AR signaling. PLCɛ up-regulation could be both a triggering factor and a supporting element during the entire process of CaP development. Further evaluations of PLCɛ/AR axis are warranted.

## Supplementary information

Supplemental Figure 1

Supplemental Figure 2

Supplemental Figure 3

Supplemental Figure 4

Supplementary figure legends

Supplemental tables
