# Supplementary figures and images for: PLCɛ maintains the functionality of AR signaling in prostate cancer via an autophagy-dependent mechanism
Source: Cell Death Dis. 2020 Sep 2;11(8):716. doi: 10.1038/s41419-020-02917-9 (PMC7468107; doi:10.1038/s41419-020-02917-9)

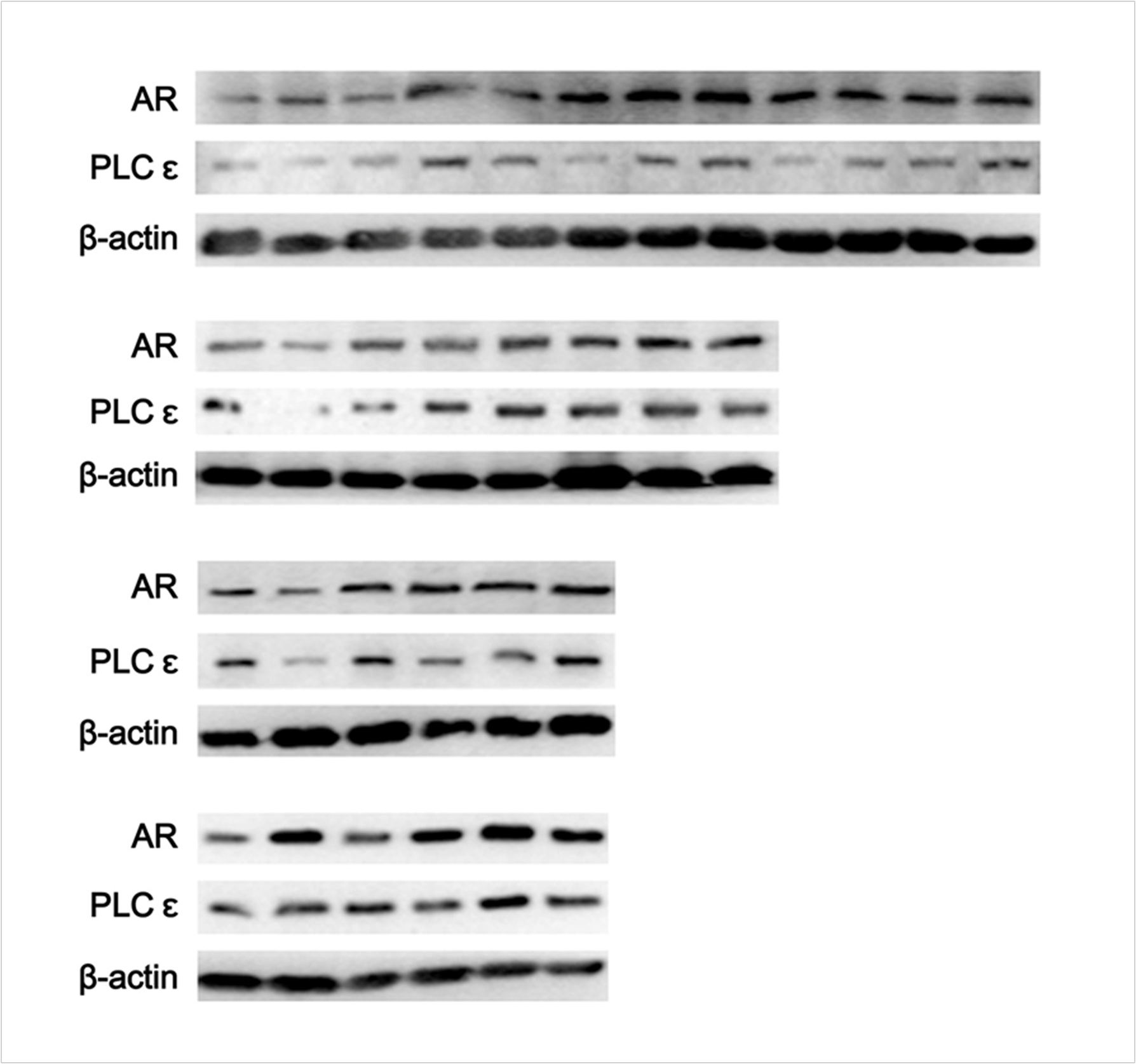

Supplement: Supplementary file 1 — Supplemental Figure 1 [file 41419_2020_2917_MOESM1_ESM.tif]

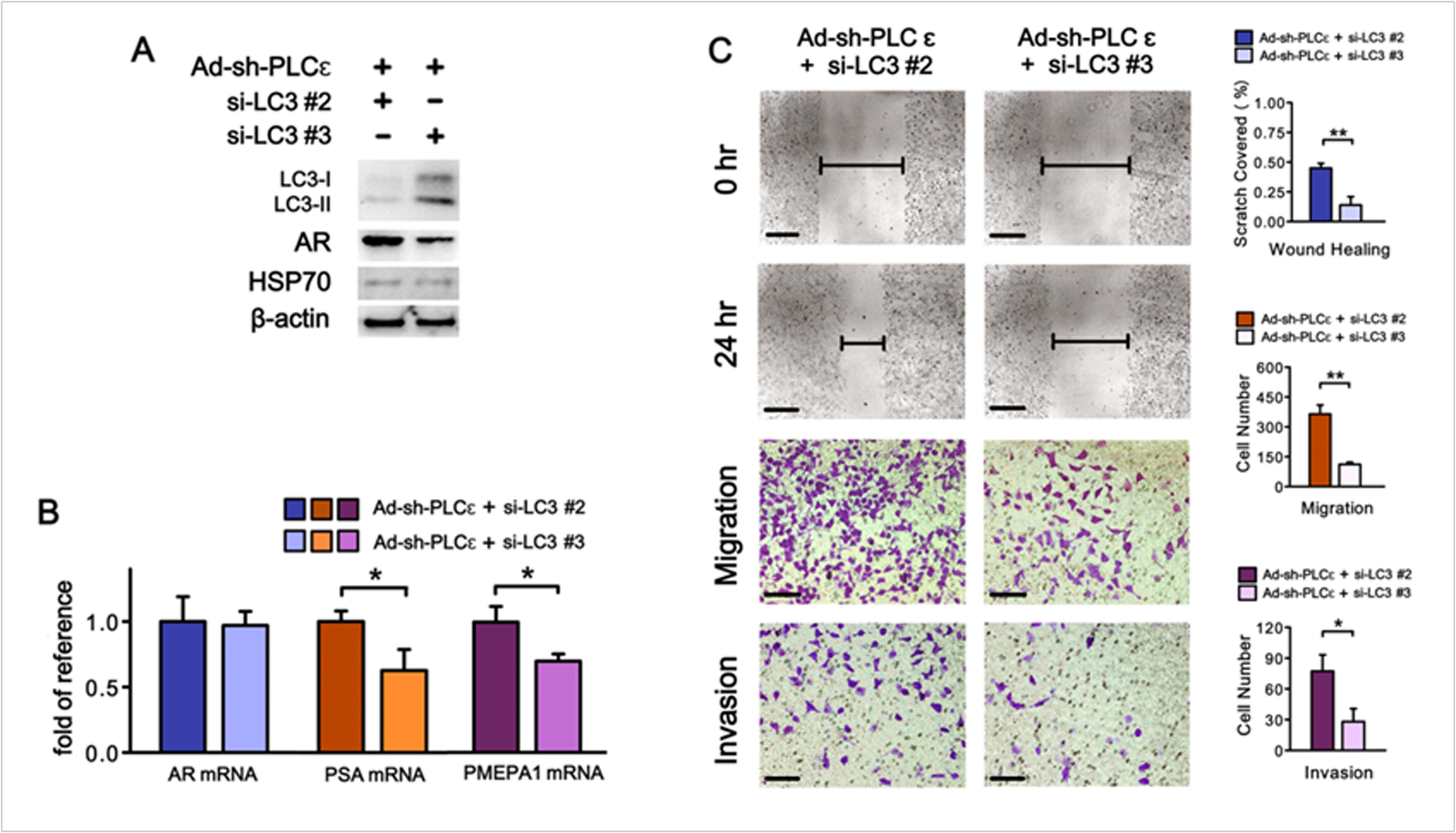

Supplement: Supplementary file 2 — Supplemental Figure 2 [file 41419_2020_2917_MOESM2_ESM.tif]

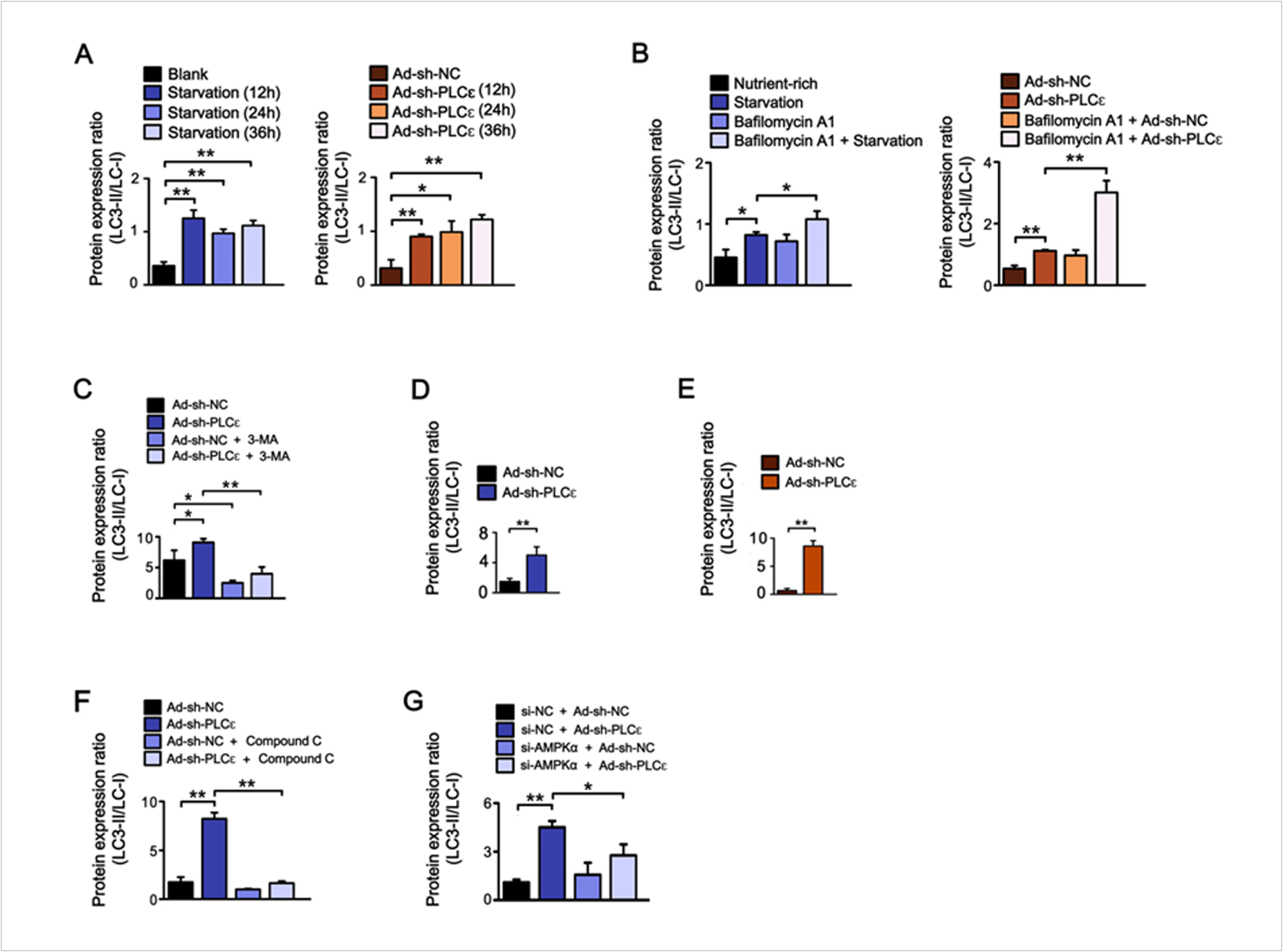

Supplement: Supplementary file 3 — Supplemental Figure 3 [file 41419_2020_2917_MOESM3_ESM.tif]

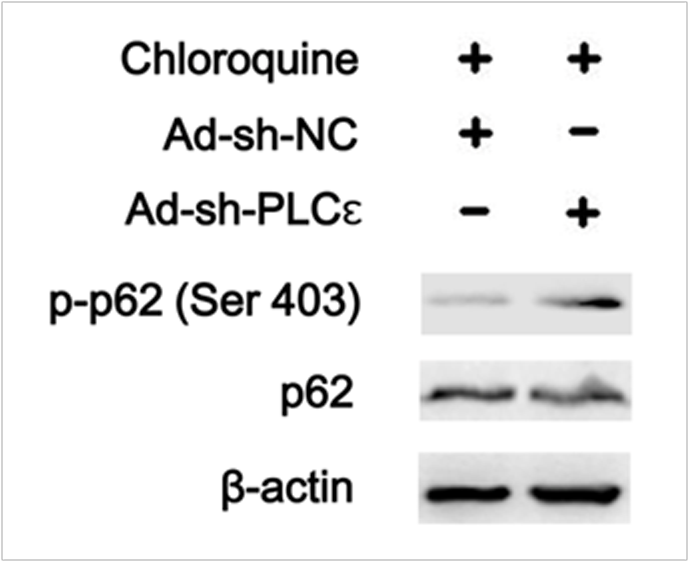

Supplement: Supplementary file 4 — Supplemental Figure 4 [file 41419_2020_2917_MOESM4_ESM.tif]
