# Supplementary material for: PLCɛ maintains the functionality of AR signaling in prostate cancer via an autophagy-dependent mechanism
Source: Cell Death Dis. 2020 Sep 2;11(8):716. doi: 10.1038/s41419-020-02917-9 (PMC7468107; doi:10.1038/s41419-020-02917-9)
Supplement: Supplementary file 5 — Supplementary figure legends [file 41419_2020_2917_MOESM5_ESM.docx]

**SFigure 1.** Protein expressions of PLCɛ and AR in 32 CaP samples.

**SFigure 2.** Effect of si-LC3 #2 and si-LC3 #3. Protein expressions of LC3-I/LC3-II, AR and HSP70 in VCaP cells subjected to PLCɛ depletion and si-LC3 #2/#3 (A). RT-PCR analysis of AR, PSA and PMEPA1 mRNA levels (B). Wound healing assay and transwell assays (C).Significance: *p < 0.05, **p < 0.01

**SFigure 3.** Quantification data of the ratio of LC3-II to LC-I in Figure 5. Significance: *p < 0.05, **p < 0.01.

**SFigure 4.** PLCɛ knockdown induced the phosphorylation of p62 at serine 403.
