# Supplementary material for: PLCɛ maintains the functionality of AR signaling in prostate cancer via an autophagy-dependent mechanism
Source: Cell Death Dis. 2020 Sep 2;11(8):716. doi: 10.1038/s41419-020-02917-9 (PMC7468107; doi:10.1038/s41419-020-02917-9)
Supplement: Supplementary file 6 — Supplemental tables [file 41419_2020_2917_MOESM6_ESM.docx]

**STable 1.** Correlation between PLCɛ and AR/hK2 in HN-CaP and Bica-R-CaP samples

|  | HN-CaP | PLCɛ | | | Kappa  value | P  value |  |  | Bica-R-CaP | PLCɛ | | | Kappa  value | P  value |
| --- | --- | --- | --- | --- | --- | --- | --- | --- | --- | --- | --- | --- | --- | --- |
|  |  | Stain  -negative | Stain  -positive | Total |  |  |  |  |  | Stain  -negative | Stain  -positive | Total |  |  |
| AR | Stain-negative | 6 | 1 | 7 | 0.570 | 0.003 |  | AR | Stain-negative | 3 | 0 | 3 | 0.824 | < 0.001 |
|  | Stain-positive | 4 | 15 | 19 |  |  |  |  | Stain-positive | 1 | 14 | 15 |  |  |
|  | Total | 10 | 16 | 26 |  |  |  |  | Total | 4 | 14 | 18 |  |  |
| hK2 | Stain-negative | 7 | 1 | 8 | 0.662 | < 0.001 |  | hK2 | Stain-negative | 2 | 0 | 2 | 0.609 | 0.005 |
|  | Stain-positive | 3 | 15 | 18 |  |  |  |  | Stain-positive | 2 | 14 | 16 |  |  |
|  | Total | 10 | 16 | 26 |  |  |  |  | Total | 4 | 14 | 18 |  |  |

^P values were calculated using the Cohen's kappa coefficient.^

**STable 2** Correlations between metastasis-related molecules and PLCɛ/AR/hK2 in HN-CaP and Bica-R-CaP samples

|  | HN-CaP | | |  | Bica-R-CaP | | |
| --- | --- | --- | --- | --- | --- | --- | --- |
| E-cadherin decrease  versus increase of | PLCɛ | AR | hK2 |  | PLCɛ | AR | hK2 |
| Kappa value | 0.420 | 0.103 | 0.262 |  | 0.375 | 0.471 | 0.217 |
| P value | 0.031 | 0.592 | 0.149 |  | 0.044 | 0.026 | 0.316 |
| Vimentin increase  versus increase of | PLCɛ | AR | hK2 |  | PLCɛ | AR | hK2 |
| Kappa value | 0.435 | 0.503 | 0.196 |  | 0.727 | 0.571 | 0.400 |
| P value | 0.009 | 0.007 | 0.178 |  | <0.001 | 0.007 | 0.034 |
| N-cadherin increase  versus increase of | PLCɛ | AR | hK2 |  | PLCɛ | AR | hK2 |
| Kappa value | 0.601 | 0.669 | 0.755 |  | 0.852 | 0.684 | 0.491 |
| P value | 0.002 | <0.001 | <0.001 |  | <0.001 | 0.002 | 0.016 |

^P values were calculated using the Cohen's kappa coefficient.^

**STable 3.** Details of STable 2.

|  | HN-CaP | E-cadherin | | | Kappa  value | P  value |  |  | Bica-R-CaP | E-cadherin | | | Kappa  value | P  value |
| --- | --- | --- | --- | --- | --- | --- | --- | --- | --- | --- | --- | --- | --- | --- |
|  |  | Stain  - positive | Stain  -negative | Total |  |  |  |  |  | Stain  - positive | Stain  -negative | Total |  |  |
| PLCɛ | Stain-negative | 6 | 4 | 10 | 0.420 | 0.031 |  | PLCɛ | Stain-negative | 2 | 2 | 4 | 0.375 | 0.044 |
|  | Stain-positive | 3 | 13 | 16 |  |  |  |  | Stain-positive | 2 | 12 | 14 |  |  |
|  | Total | 9 | 17 | 26 |  |  |  |  | Total | 4 | 14 | 18 |  |  |
| AR | Stain-negative | 3 | 4 | 7 | 0.103 | 0.592 |  | AR | Stain-negative | 2 | 1 | 3 | 0.471 | 0.026 |
|  | Stain-positive | 6 | 13 | 19 |  |  |  |  | Stain-positive | 2 | 13 | 15 |  |  |
|  | Total | 9 | 17 | 26 |  |  |  |  | Total | 4 | 14 | 18 |  |  |
| hK2 | Stain-negative | 4 | 4 | 8 | 0.262 | 0.149 |  | hK2 | Stain-negative | 1 | 1 | 2 | 0.217 | 0.316 |
|  | Stain-positive | 5 | 13 | 18 |  |  |  |  | Stain-positive | 3 | 13 | 16 |  |  |
|  | Total | 9 | 17 | 26 |  |  |  |  | Total | 4 | 14 | 18 |  |  |

|  | HN-CaP | Vimentin | | | Kappa  value | P  value |  |  | Bica-R-CaP | Vimentin | | | Kappa  value | P  value |
| --- | --- | --- | --- | --- | --- | --- | --- | --- | --- | --- | --- | --- | --- | --- |
|  |  | Stain  - negative | Stain  -positive | Total |  |  |  |  |  | Stain  - negative | Stain  - positive | Total |  |  |
| PLCɛ | Stain-negative | 10 | 0 | 10 | 0.435 | 0.009 |  | PLCɛ | Stain-negative | 4 | 0 | 4 | 0.727 | <0.001 |
|  | Stain-positive | 8 | 8 | 16 |  |  |  |  | Stain-positive | 2 | 12 | 14 |  |  |
|  | Total | 18 | 8 | 26 |  |  |  |  | Total | 6 | 12 | 18 |  |  |
| AR | Stain-negative | 6 | 1 | 7 | 0.503 | 0.007 |  | AR | Stain-negative | 3 | 0 | 3 | 0.571 | 0.007 |
|  | Stain-positive | 5 | 14 | 19 |  |  |  |  | Stain-positive | 3 | 12 | 15 |  |  |
|  | Total | 11 | 15 | 26 |  |  |  |  | Total | 6 | 12 | 18 |  |  |
| hK2 | Stain-negative | 7 | 1 | 8 | 0.196 | 0.178 |  | hK2 | Stain-negative | 2 | 0 | 2 | 0.400 | 0.034 |
|  | Stain-positive | 11 | 7 | 18 |  |  |  |  | Stain-positive | 4 | 12 | 16 |  |  |
|  | Total | 18 | 8 | 26 |  |  |  |  | Total | 6 | 12 | 18 |  |  |

|  | HN-CaP | N-cadherin | | | Kappa  value | P  value |  |  | Bica-R-CaP | N-cadherin | | | Kappa  value | P  value |
| --- | --- | --- | --- | --- | --- | --- | --- | --- | --- | --- | --- | --- | --- | --- |
|  |  | Stain  - negative | Stain  -positive | Total |  |  |  |  |  | Stain  - negative | Stain  - positive | Total |  |  |
| PLCɛ | Stain-negative | 8 | 2 | 10 | 0.601 | 0.002 |  | PLCɛ | Stain-negative | 4 | 0 | 4 | 0.852 | <0.001 |
|  | Stain-positive | 3 | 13 | 16 |  |  |  |  | Stain-positive | 1 | 13 | 14 |  |  |
|  | Total | 11 | 15 | 26 |  |  |  |  | Total | 5 | 13 | 18 |  |  |
| AR | Stain-negative | 7 | 0 | 7 | 0.669 | <0.001 |  | AR | Stain-negative | 3 | 0 | 3 | 0.684 | 0.002 |
|  | Stain-positive | 4 | 15 | 19 |  |  |  |  | Stain-positive | 2 | 13 | 15 |  |  |
|  | Total | 11 | 15 | 26 |  |  |  |  | Total | 5 | 13 | 18 |  |  |
| hK2 | Stain-negative | 8 | 0 | 8 | 0.755 | <0.001 |  | hK2 | Stain-negative | 2 | 0 | 2 | 0.491 | 0.016 |
|  | Stain-positive | 3 | 15 | 18 |  |  |  |  | Stain-positive | 3 | 13 | 16 |  |  |
|  | Total | 11 | 15 | 26 |  |  |  |  | Total | 5 | 13 | 18 |  |  |

^P values were calculated using the Cohen's kappa coefficient.^

**STable 4.** Prostate Tissue Samples in the First Section of the Study

| Sample Type | Surgical Procedure | Amount | Chemotherapy or ADT |
| --- | --- | --- | --- |
| CaP | Radical Prostatectomy | 55 | No |
| CaP | Prostate Biopsy | 37 | No |
| BPH | TURP | 42 | No |
| BPH | Radical Cystectomy (for bladder cancer) | 24 | No |
| NP | Radical Cystectomy (for bladder cancer) | 10 | No |

^Normal Prostate, NP; Benign Prostatic Hyperplasia, BPH; Prostate Cancer, CaP; Transurethral Resection of the Prostate, TURP.^

^All patients selected were free of other prostate-related disease and had not received endocrine therapies that could potential influence AR signaling.^

**STable 5.** Prostate Tissue Samples in the Last Section of the Study

| Sample Type | Surgical Procedure | Amount | Chemotherapy or ADT |
| --- | --- | --- | --- |
| HN-CaP | Radical Prostatectomy | 26 | No |
| Bica-R-CaP | Palliative TURP  (to relieve severe symptoms of LUTS) | 18 | Bicalutamide treatment |

^HN-CaP, Hormone-Naive Prostate Cancer : Samples from patients receiving no ADT or other anti-androgen therapies.^

^Bica-R-CaP, Bicalutamide-Resistant Prostate Cancer: Bicalutamide resistance is defined as the occurrence of CRPC during bicalutamide treatment (patients receiving simultaneous ADT and anti-androgen therapies were excluded).^

**STable 6.** Antibodies Used in Immunohistochemistry

| Primary antibody | Dilution | Source |
| --- | --- | --- |
| Rabbit polyclonal PLCɛ | 1:500 | Santa Cruz, US |
| Mouse monoclonal AR | 1:300 | Santa Cruz, US |
| Rabbit polyclonal hK2 | 1:500 | Thermo Fisher, US |
| Mouse monoclonal E-cadherin | 1:300 | Santa Cruz, US |
| Rabbit monoclonal Vimentin | 1:500 | Abcam, US |
| Rabbit polyclonal N-cadherin | 1:500 | Abcam, US |

**STable 7.** Algorithm of Semi-quantitative Scoring in Immunohistochemistry

| Staining Intensity | Score | Immunoreactive Cells | Score |
| --- | --- | --- | --- |
| No Staining | 0 | 0% | 0 |
| Light Yellow | 1 | <5% | 1 |
| Light Brown | 2 | 5–50% | 2 |
| Brown | 3 | >5% | 3 |

^The final staining score was defined as the sum of both parameters.^

^Clinical samples were grouped as negative/weak (0–2), moderate (3) and strong (4–6) staining.^

**STable 8.** Sources of cell lines and culture media.

| Cell line | Source | Media | Source |
| --- | --- | --- | --- |
| RWPE-1 | ATCC, US | K-SFM | Gibco, US |
| LNCaP | ATCC, US | RPMI-1640 | Gibco, US |
| VCaP | ATCC, US | DMEM/F-12 | Gibco, US |
| C4-2B | ATCC, US | DMEM/F-12 | Gibco, US |
| PC3 | ATCC, US | DMEM/F-12 | Gibco, US |
| DU145 | ATCC, US | DMEM/F-12 | Gibco, US |
| 22RV1 | Cell Bank of Type Culture Collection of the Chinese Academy of Sciences, China | RPMI-1640 | Gibco, US |

^CaP cell lines were maintained in corresponding media, supplemented with 10% fetal bovine serum (Gibco, US).^

^RWPE-1 was supplemented with 0.05 mg/ml BPE and 5 ng/ml EGF (Gibco, US).^

^EBSS was also purchased from Gibco.^

^KRH buffer was prepared as described [32].^

**STable 9.** Sources of Small Interfering RNAs.

| siRNA | Catalog | Source |
| --- | --- | --- |
| si-LC3/MAP1LC3B | s37748 | Invitrogen |
| si-p62/SQSTM1 | s16961 | Invitrogen |
| si-NBR1 | s227479 | Invitrogen |
| si-AMPKα/PRKAA1 | s100 | Invitrogen |

**STable 10.** Sources of reagents

| Reagent | Source |
| --- | --- |
| bicalutamide | Selleck, US |
| enzalutamide | Selleck, US |
| DHT/dihydrotestosterone | Sigma-Aldrich, US |
| cycloheximide | MedchemExpress, US |
| MG132 | MedchemExpress, US |
| ALLN | MedchemExpress, US |
| bafilomycin A1 | Selleck, US |
| 3-MA/3-methyladenine | Selleck, US |
| compound C | Selleck, US |
| PMA/phorbol 12-myristate 13-acetate | MedchemExpress, US |
| chloroquine | Sigma-Aldrich, US |

**STable 11.** Primers for RT-PCR

| Genes | Primer Sequences | | | |
| --- | --- | --- | --- | --- |
|  | Forward | | Reverse | |
| PLCE1 (PLCɛ) | | CATGGAAGGATAAGCGTTGGT | | CCCAAGTCCCGTGTTAAGA |
| AR | | CAGTGGATGGGCTGAAAAAT | | GGAGCTTGGTGAGCTGGTAG |
| KLK3 (PSA) | | ACGCTGGACAGGGGGCAAAAG | | GGGCAGGGCACATGGTTCACT |
| PMEPA 1 | | CATGATCCCCGAGCTGCT | | TGATCTGAACAAACTCCAGCTCC |
| MAP1LC3B (LC3) | | GACCGCTGTAAGGAGGTGC | | CTTGACCAACTCGCTCATGTTA |
| BECN1 (Beclin 1) | | ACTGTGTTGCTGCTCCATGC | | CCCAAGCAAGACCCCACTTA |
| SQSTM1 (p62) | | GGGGACTTGGTTGCCTTTT | | CAGCCATCGCAGATCACATT |
| ULK 1 | | ACGACTTCCAGGAAATGGCTA | | GGAAGAGCCTGATGGTGTCCT |
| ACTB (β-actin) | | TGACGTGGACATCCGCAAAG | | CTGGAAGGTGGACAGCGAGG |

**STable 12.** Antibodies Used in Western Blot

| Primary antibodies | Dilution | Source |
| --- | --- | --- |
| Rabbit polyclonal PLCɛ | 1:1000 | Santa Cruz, US |
| Rabbit monoclonal AR | 1:2000 | Cell Signaling Technology, US |
| Mouse monoclonal PSA | 1:1000 | Santa Cruz, US |
| Mouse monoclonal E-cadherin | 1:1000 | Santa Cruz, US |
| Rabbit polyclonal N-cadherin | 1:2000 | Abcam, US |
| Mouse monoclonal Vimentin | 1:1000 | Santa Cruz, US |
| Rabbit monoclonal LC3-I/II | 1:2000 | Novus, US |
| Rabbit polyclonal HSP 70 | 1:1000 | ProteinTech, US |
| Mouse monoclonal Beclin 1 | 1:1000 | Cell Signaling Technology, US |
| Rabbit polyclonal ULK 1 | 1:2000 | Sigma-Aldrich, US |
| Rabbit monoclonal phospho-ULK 1 ser 555 | 1:1000 | Cell Signaling Technology, US |
| Rabbit polyclonal phospho-ULK 1 ser 757 | 1:1000 | Cell Signaling Technology, US |
| Rabbit monoclonal p62 | 1:2000 | Abcam, US |
| Rabbit monoclonal phospho-p62 ser 403 | 1:1000 | Cell Signaling Technology, US |
| Rabbit polyclonal VPS34 | 1:2000 | Novus, US |
| Rabbit polyclonal AKT | 1:1000 | ProteinTech, US |
| Mouse monoclonal phospho-AKT ser 473 | 1:1000 | Santa Cruz, US |
| Rabbit polyclonal mTOR | 1:1000 | Santa Cruz, US |
| Rabbit monoclonal phospho-mTOR ser 2448 | 1:1000 | Cell Signaling Technology, US |
| Rabbit monoclonal AMPKα | 1:2000 | Cell Signaling Technology, US |
| Rabbit monoclonal phospho-AMPKα thr 172 | 1:1000 | Cell Signaling Technology, US |
| Rabbit monoclonal phospho-AMPKα ser 485 | 1:1000 | Cell Signaling Technology, US |
| Rabbit monoclonal NBR1 | 1:2000 | Cell Signaling Technology, US |
| Rabbit polyclonal β-actin | 1:1000 | ProteinTech, US |
| Rabbit polyclonal Histone H3 | 1:2000 | ProteinTech, US |

^Total protein of tissue samples or cells was extracted using RIPA buffer (Beyotime, China) supplemented with protease inhibitor PMSF (Beyotime, China) and phosphatase inhibitors Na3VO4 (Sigma, US).^

^Nuclear and cytoplasmic proteins were extracted separately using the Nuclear and Cytoplasmic Protein Extraction Kit (Beyotime, China).^

**STable 13.** Antibodies used in Immunofluorescence

| Primary antibodies | Dilution | Source |
| --- | --- | --- |
| Mouse monoclonal AR | 1:300 | Santa Cruz, US |
| Rabbit monoclonal NBR1 | 1:800 | Cell Signaling Technology, US |
| Rabbit monoclonal p62 | 1:500 | Abcam, US |
| Rabbit monoclonal LC3-I/II, | 1:500 | Novus, US |
| Secondary antibodies | Dilution | Source |
| FITC goat anti-rabbit IgG | 1:200 | ZSGB-BIO, China |
| TRITC goat anti-mouse IgG | 1:200 | ZSGB-BIO, China |

**STable 14.** Antibodies used in Immunoprecipitation

| Primary antibodies | Concentration | Source |
| --- | --- | --- |
| Mouse monoclonal AR | 1 μg antibody  per 1 mg protein | Santa Cruz, US |
| Mouse monoclonal NBR1 |  | Abcam, US |
| Mouse monoclonal p62 |  | Santa Cruz, US |
